# Generation of a synthetic GlcNAcylated nucleosome reveals regulation of stability by H2A-Thr101 GlcNAcylation

**DOI:** 10.1038/ncomms8978

**Published:** 2015-08-25

**Authors:** Lukas Lercher, Ritu Raj, Nisha A. Patel, Joshua Price, Shabaz Mohammed, Carol V. Robinson, Christopher J. Schofield, Benjamin G. Davis

**Affiliations:** 1 Department of Chemistry, University of Oxford, Chemistry Research Laboratory, Mansfield Road, Oxford OX1 3TA, UK; 2 Department of Chemistry, University of Oxford, Physical and Theoretical Chemistry Laboratory, South Parks Road, Oxford OX1 3QZ, UK

## Abstract

*O-*GlcNAcylation is a newly discovered histone modification implicated in transcriptional regulation, but no structural information on the physical effect of GlcNAcylation on chromatin exists. Here, we generate synthetic, pure GlcNAcylated histones and nucleosomes and reveal that GlcNAcylation can modulate structure through direct destabilization of H2A/H2B dimers in the nucleosome, thus promoting an ‘open' chromatin state. The results suggest that a plausible molecular basis for one role of histone *O*-GlcNAcylation in epigenetic regulation is to lower the barrier for RNA polymerase passage and hence increase transcription.

## Abstract (web summary)

Post-translational modification of histones has been implicated in gene regulation. Here, Lercher *et al.* generate synthetic GlcNAcylated histone 2A and nucleosomes and show that this modification can cause nucleosome destabilization, suggesting histone 2A *O*-GlcNAcylation may promote an open chromatin state and increase transcription.

## Graphical abstract

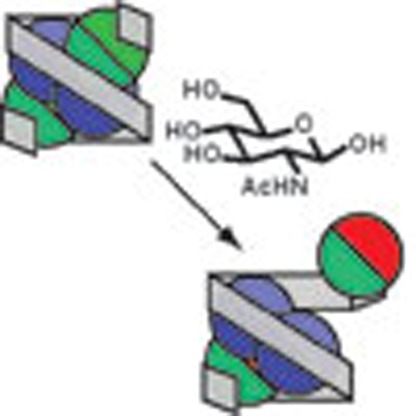

The post-translational modification (PTM) of histones is a widespread mechanism for the regulation of eukaryotic gene expression^1^. Addition of *O*-linked β-*N*-acetylglucosamine (GlcNAc) to serine- and threonine-residues is an abundant PTM of cytosolic and nuclear proteins^2^ and has recently been identified on histones^3^,^4^. *O*-GlcNAc sites have been detected on all canonical histones, with H2B-Ser112 *O*-GlcNAcylation being linked to transcriptional activation via promotion of H2B ubiquitinylation^5^. More generally, protein *O*-GlcNAcylation functions in nutrient sensing and is essential for development^6^.

In mammals, *O*-GlcNAcylation is catalysed by a single enzyme, *O*-linked *N*-acetylglucosamine transferase (OGT). OGT interacts both with transcriptionally repressing^7^ and activating^8^ histone modifying complexes suggesting a context dependent role for OGT in transcriptional regulation, as occurs with other histone PTMs, for example, acetylation, methylation and phosphorylation. To date, no molecular mechanism for the transcriptional regulation by histone *O*-GlcNAcylation has been reported. To define the molecular roles of OGT in transcriptional regulation, access to homogeneously GlcNAcylated histones is required. Homogeneous *O*-GlcNAcylation is not attainable enzymatically because OGT is promiscuous^9^; *in vitro* reactions on histones yield mixtures of *O*-GlcNAcylated products^5^ and attaining complete PTM is difficult. Here, we report the selective synthesis of a nucleosome containing histone H2A bearing a single *O*-GlcNAc-mimic at threonine (Thr) 101, a functionally unassigned *O*-GlcNAcylation site. Nucleosomes contain an octameric core, in which two H2A/B dimers interact on opposite sides of an H3/H4 tetramer. We show here that H2A-Thr101 GlcNAcylation reduces nucleosome stability through destabilization of the H3/H4 tetramer—H2A/B dimer interface. On the basis of these results we suggest that regulation of nucleosome stability by OGT-dependent GlcNAc transfer may contribute to transcriptional regulation.

## Results

### Generation of homogeneous GlcNAcylated H2A

**Figure 1:**
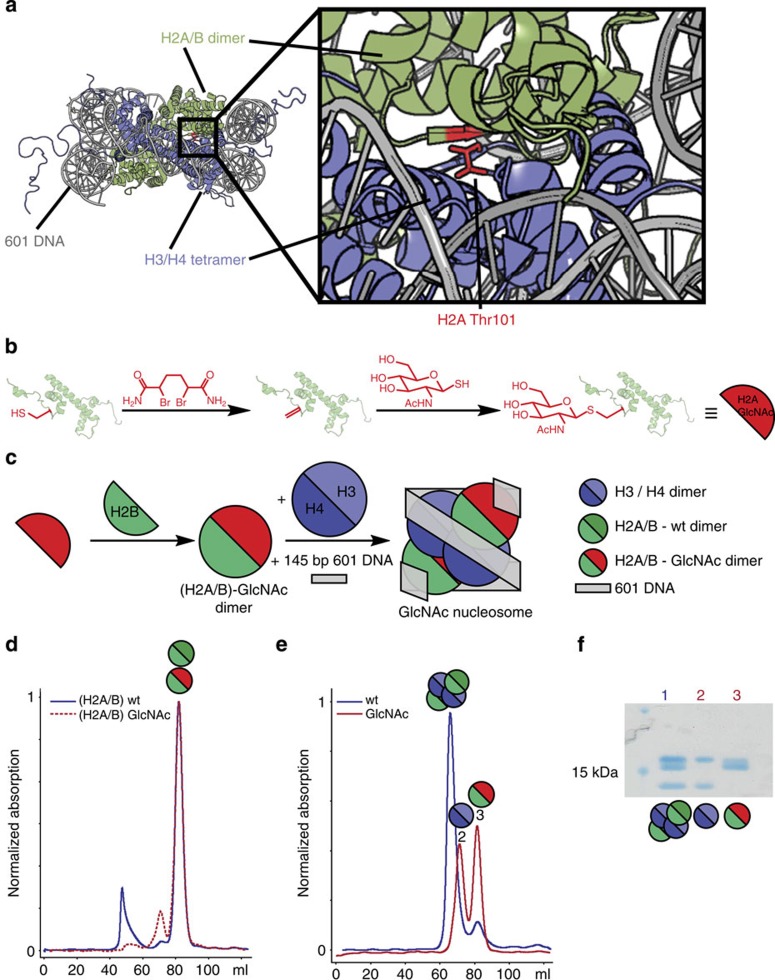
Modification and refolding of nucleosome complexes with H2A/B-GlcNAc. (**a**) Graphic representation of a nucleosome structure with H2A Thr-101 in red (adapted from 1KX4)^51^. (**b**) Reaction scheme for generation of GlcNAcylated H2A. Cys-101 is converted to dehydroalanine (Dha), which functions as a Michael acceptor for reaction with the GlcNAc-thiol, so replacing the ether linkage with a structurally analogous thioether. (**c**) Schematic representation of H2A/B dimer and nucleosome refolding (wt H2A in dark green and GlcNAcylated H2A in red). (**d**) SEC traces for the refolding of H2A/B dimer from wt (blue) and H2A T101GlcNAc (red) and wt H2B. (**e**) SEC traces for the octamer refolding from wt H3/H4 tetramers and H2A/B-wt (blue) or H2A/B-GlcNAc (red) dimers. (**f**) SDS–PAGE analysis of the main fractions of the marked peaks (original image shown in Supplementary Fig. 16).

We have reported a chemical PTM strategy^10^,^11^, that enables generation of homogenously GlcNAcylated proteins including histone H3. Of the few reported *in vivo O*-GlcNAcylation sites in nucleosomes, histone H2A-Thr101 (refs 3, 5) is at the H2A/B dimer and H3/H4 tetramer interface (Fig. 1a). Transcriptional regulation by PTMs is often achieved through selective binding to PTMs by effector proteins^1^. Since no selective *O*-GlcNAc-histone-binding proteins have been reported, we wanted to explore if GlcNAcylation could function as an epigenetic signal by directly affecting nucleosome structure. Thr101 modification could potentially influence nucleosome stability, by modulating dimer/tetramer association. To date, no functional consequences of H2A-Thr101 modification have been reported.

To generate homogenous GlcNAcylated nucleosomes we produced and purified a recombinant Thr101Cys variant of the *Xenopus laevis* H2A (Supplementary Fig. 1)^12^. H2A-T101C was treated with 2,5-dibromohexanediamide (DBHDA) at 37 °C for 3 h under denaturing conditions (5 M guanidine.HCl) to yield a dehydroalanine (Dha) residue at H2A-101 after di-alkylation and elimination (Fig. 1b and Supplementary Fig. 2). Following removal of excess DBHDA by desalting, GlcNAc-thiol was reacted with the Dha containing protein to yield the first homogenous H2A containing a *O*-GlcNAc-mimic at residue 101 (Fig. 1b and Supplementary Fig. 3), as shown by liquid chromatography-mass spectrometry (LC-MS). We then used the GlcNAcylated H2A to produce modified nucleosomes in a stepwise manner, that is, by initial formation of H2A/B dimers, followed by addition of the H3/H4 tetramer and the high affinity 601 DNA sequence, to give folded nucleosomes (Fig. 1c).

### H2A-T101 GlcNAcylation reduces tetramer-dimer association

To investigate the effect of GlcNAcylation we analysed intermediates by size-exclusion chromatography (SEC) and circular dichroism (CD) spectroscopy. SEC gives information on size and homogeneity and is used to purify histone octamers or sub-complexes for nucleosome reconstitution^12^, while CD allows comparison of secondary structure content and stability of complexes. H2A/B dimers were constructed using equimolar amounts of histones and then analysed and purified by SEC^12^. Identical SEC traces for (H2A/B)-wt (wild type) and (H2A/B)-GlcNAc indicate that H2A-101 GlcNAcylation does not substantially influence viable dimerization (Fig. 1d). The conservation of dimer structure and stability was further confirmed by variable temperature CD analysis (Supplementary Fig. 4). The obtained melting temperatures were similar with and without GlcNAcylation ((H2A/B)-GlcNAc, 50.7±0.1 °C; (H2A/B)-wt, 52.9±0.1 °C)^13^. Together these results suggest that H2A-101 GlcNAcylation also does not significantly influence H2A/B dimer structure and stability.

Next, synthetic histone octamers were examined. To refold octamers for nucleosome reconstitution, we combined (H2A/B)-wt and (H2A/B)-GlcNAc with a purified wt H3/H4 tetramer (Supplementary Fig. 4d) and analysed the resulting mixture by SEC. Although for the (H2A/B)-wt we observed octamer formation as anticipated, for the (H2A/B)-GlcNAc we observed only separately eluting di- and tetramers, suggesting GlcNAcylation destabilizes the octamer (Fig. 1e,f). The potential exists for the creation of D- and L-epimers at Cα of the GlcNAcylation site. To verify that observed destabilization was not due to an artefact resulting only from differing amino acid stereochemistry, we incubated the H3/H4 tetramers with an excess of modified H2A/B dimers (1.5- and 2-fold) and separated the formed complexes by SEC (Supplementary Fig. 5). Again we observed no octamer formation, but separately eluting di- and tetramers, consistent with destabilization regardless of chirality.

### H2A-T101 GlcNAcylation decreases nucleosome stability

**Figure 2:**
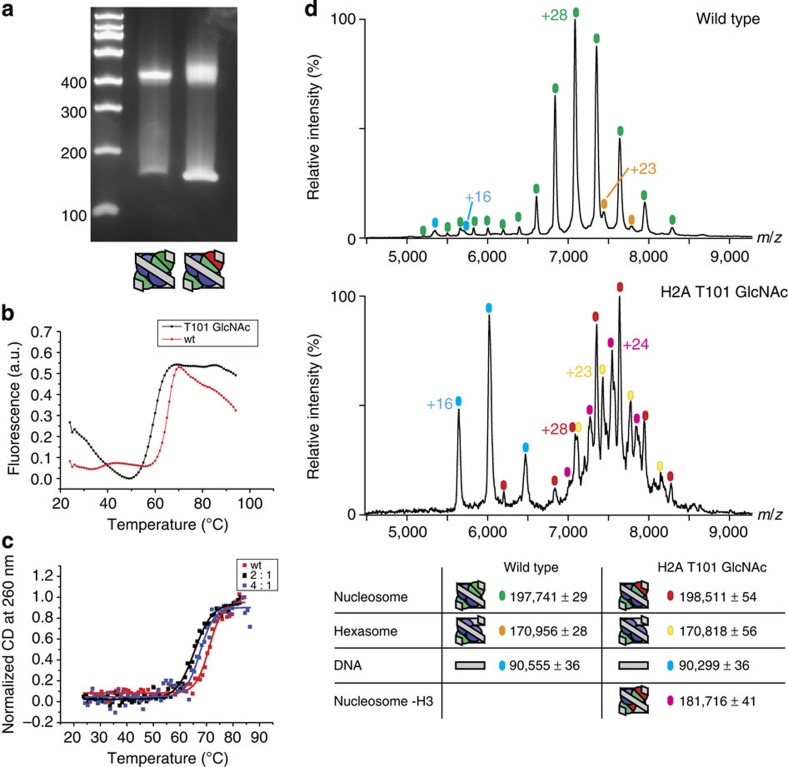
Comparison of stability and composition of nucleosomes refolded using H2A/H2B-wt or H2A/H2B-GlcNAc dimers and wt H3/H4 tetramers. (**a**) 6% TBE-PAGE gel analysis of salt gradient refolding of wt (left) and GlcNAcylated (right) nucleosomes. (**b**) Melting profile of wild type and H2A Thr-101 GlcNAcylated nucleosome as assayed by DSF. Experiments were performed in duplicates. (**c**) Melting profile of wild type and H2A Thr-101 GlcNAcylated nucleosomes reconstituted with different dimer: tetramer ratios (2:1 and 4:1) as monitored by CD at 260 nm. (**d**) Non-denaturing ESI-MS analysis of wt and GlcNAcylated nucleosomes. Values reported represent the mean value and standard deviation of the observed m/z peaks.

In cells histones do not exist as free octamers but are complexed by chaperones or DNA and chromatin-associated proteins^14^. To investigate the effect of GlcNAcylation on complete nucleosome structure, we refolded nucleosomes from separately purified H3/H4 tetramers and the (H2A/B)-GlcNAc dimers using the salt gradient method^12^ and a 145 bp DNA construct^15^. We used the selective and tightly binding ‘601' nucleosome positioning sequence to obtain homogeneous nucleosomes. Using PAGE, we observed similarly migrating bands for the wt nucleosome reconstituted from wt histone octamers and the GlcNAcylated nucleosome, although the band for the GlcNAc-modified nucleosome appeared more diffuse (Fig. 2a). Again, to control for potential artefacts from stereochemistry at the GlcNAcylation site, we reconstituted nucleosomes with different tetramer/dimer ratios (1.5–5 eq dimer). PAGE analysis revealed similarly migrating species for all reconstitutions (Supplementary Fig. 6). To further probe the structure of these nucleosomes we used varying salt concentrations; it has been proposed that when ionic strength is elevated, non-native species dissociate at lower ionic strength than canonical nucleosomes^16^,^17^. When incubated at intermediate ionic strength (up to 600 mM KCl; Supplementary Fig. 7) both the wt and GlcNAcylated nucleosomes remained stable, indicating a native structure similar to canonical nucleosomes for both species that does not stem from unspecific non-native binding of histones to DNA.

To investigate if the H2A-101 GlcNAcylation destabilises nucleosomes, we measured the thermal stability of both wt and GlcNAcylated nucleosome by differential scanning fluorimetry^18^ (DSF), CD and electrophoretic analysis (Fig. 2b,c, Supplementary Figs 8–10). The DSF measurements revealed a significant reduction in melting temperature for the GlcNAcylated nucleosomes (59.6 °C (GlcNAc) versus 65.6 °C (wt)). CD analyses also supported the significant destabilization of the H2A-101GlcNAc-containing nucleosome. Although DSF reports on protein melting, by using CD both the protein and/or DNA structural transitions of the nucleosome dissociation process can be measured^19^. The CD spectrum above 250 nm originates almost exclusively from the DNA and is sensitive to structure. The CD spectra of the unmelted wt and the GlcNAcylated nucleosomes (2:1 dimer:tetramer) are near identical (Supplementary Fig. 8); at higher dimer concentrations (4:1 dimer:tetramer) the observed CD is increased above 240 nm suggesting slight changes in DNA structure. Monitoring the thermal dissociation profile for these wt and GlcNAcylated nucleosomes at the higher wavelength 260 nm, revealed a single transition for all complexes (Fig. 2). As for the observations made by DSF, the GlcNAcylated nucleosomes were significantly destabilized (Tm 2:1, 65.5±0.2 °C; 4:1, 67.6±0.4 °C) compared with the wt nucleosome (Tm 71.0±0.3 °C; ). When monitoring the thermal dissociation by CD at 220 nm, we observed apparent two-state melting profiles; the first transition for H2A-T101GlcNAc nucleosome was shifted to a lower Tm relative to that of the wt nucleosome (Supplementary Fig. 9, Tm 2:1, 65.8±0.9 °C; 4:1, 66.1±0.4 °C; wt, 69.7±0.3 °C). After this first transition both nucleosomes behave similarly with respect to the change in CD with temperature. These observations are consistent with a first transition that corresponds to the dissociation and denaturation of the H2A/B dimer, while the second corresponds to the dissociation and denaturation of the H3/H4 tetramer. To further investigate these dissociation pathways, we incubated wt or GlcNAcylated nucleosomes at different temperatures around the measured Tm and analysed the resulting soluble fraction by SDS–PAGE (Supplementary Fig. 10). In agreement with previous assays, we observed earlier disassembly of the GlcNAcylated nucleosome, indicated by higher migrating bands in the native PAGE (Supplementary Fig. 10a). In the soluble fractions we observed selective loss of H2A and H2B from GlcNAcylated nucleosome at lower temperatures (Supplementary Fig. 10b). These results support the proposed destabilization of the H2A/B dimer in the context of the nucleosome.

### GlcNAcylated nucleosomes consist of mixtures of subspecies

We then used nanoelectrospray ionization mass spectrometry (nESI-MS), under conditions that substantially preserve non-covalent interactions^20^, to further characterize the GlcNAcylated nucleosome complex. nESI-MS allows accurate analysis and assignment of formed nucleosome complexes. nESI-MS analysis of wt nucleosomes revealed one major set of peaks corresponding to the intact nucleosome, and lower levels of hexasome (∼16%) and free DNA (Fig. 2d). In contrast, the GlcNAcylated nucleosome (reconstituted at a 1:2 ratio of H3/H4 tetramer: H2A/H2B dimer) does not constitute a homogenous species; at least three overlapping ion series were observed (Fig. 2d). These were assigned to an intact nucleosome; a species lacking one H3 subunit and a hexasome lacking one H2A/B dimer. Although observation of a hexasome has previously been reported^20^, the seven subunit containing species lacking one histone H3 has not been previously described. To test whether the observed species were possible artefacts of nucleosome reconstitution from separated H2A/B dimers and H3/H4 tetramers, we also reconstituted wt nucleosome from mixed wt H2A/B dimers and H3/H4 tetramers (Supplementary Fig. 11). These displayed a near identical behaviour when analysed by non-denaturing gel electrophoresis, CD and nESI-MS. We also analysed GlcNAcylated nucleosomes reconstituted with increasing concentrations of H2A/H2B dimer (up to 4.5 eq with respect to the H3/H4 tetramer) by nESI-MS (Supplementary Fig. 12). Although the relative amount of sub-complexes varied with H2A/H2B equivalents, all samples showed decreased amount of intact nucleosome when compared with the wt sample. In wt nucleosome structures the histone H3 α-3 helix is located close to the modified H2A-101 site. It is possible that a steric clash between this helix and the H2A-T101GlcNAc results in preferred formation of species lacking either one H2A/H2B dimer or one histone H3. Together these results support the proposal that *O*-GlcNAcylation at the H2A-101 site destabilizes nucleosomes.

**Figure 3:**
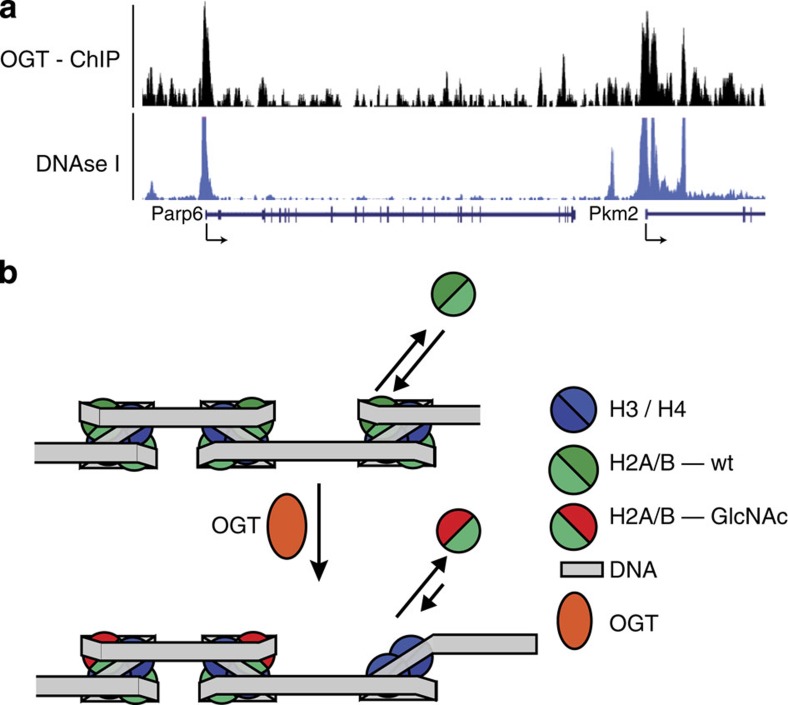
Analysis of OGT function. (**a**) Genomic snapshots from the UCSC genome browser for OGT ChIP-Seq^21^ and DNaseI Hypersensitivity by Digital DNaseI from ENCODE/University of Washington (ES-E14 cells)^22^ in mouse embryonic stem cells. (**b**) Model for generation and maintenance of open chromatin states by OGT recruitment. When OGT is present (possibly through OGT recruitment, see Supplementary Fig. 15) H2A Thr-101 can be *O*-GlcNAcylated so causing destabilization of the H2A/H2B dimer in the nucleosome. This promotes hexasome formation and generation of an open chromatin structure.

These combined biophysical studies of GlcNAcylated nucleosomes suggest that PTM mediated by OGT at H2A-T101 may regulate nucleosome stability *in vivo* via modulation of H2A/B dimer/tetramer interactions. This proposal was analysed further by comparison of identified sites of open chromatin and OGT localization in the genome (Fig. 3)^21^,^22^. DNAse I sensitivity is a measure of chromatin accessibility and thereby nucleosome stability. A strong correlation with almost all identified OGT sites (97%, *P*=0.000999, Monte Carlo False Discovery rate (MCFDR)) overlapping with reported DNAse I sensitive regions (Fig. 3a) was observed. Transcription start sites (TSS) for active operons contain nucleosome depleted regions, which are DNAse I sensitive; OGT was also associated with protein complexes that regulate transcription at TSS. A strong correlation with 97% of all OGT peaks overlapping with DNAse I sensitive regions at TSS was also observed. These observations are in agreement with the generation of an open chromatin state by OGT recruitment.

### GlcNAcylation causes changes in the interactome

Transcriptional regulation by various PTM on the nucleosome involves recruitment of chromatin ‘reader' proteins^23^. To gain further insights into the role of H2A-T101 *O*-GlcNAcylation *in vivo*, we probed the effects of nucleosome GlcNAcylation upon nucleosome interaction partners from human cells. We reconstituted nucleosomes using biotinylated DNA containing the 601 sequence^24^; these were then immobilized via biotin onto streptavidin-coated beads and used to interrogate putative nucleosome-binding proteins from HeLa cell nuclear extracts, using synthetically GlcNAcylated and wt nucleosomes (‘bait' and control, respectively). The experiments were performed as two independent biological replicates. The enriched proteins were digested and analysed by nLC-MS/MS. MS-based quantitative proteomics has been extensively used to identify reader proteins for various chromatin markers^24^,^25^. Label-free quantification^26^ enabled us to identify 20 proteins, among other identified proteins (Supplementary Data 1), which were over threefold enriched in the GlcNAcylated nucleosome sample (Supplementary Fig. 13); of these 12 (60%) can bind to DNA. Notably, these included USF1 and USF2 that bind an E-Box sequence^27^ present in the DNA sequence used here. Inspection of nucleosome crystal structures^15^ shows that this region is normally bound by the H2A/B dimer (Supplementary Fig. 14). Additionally, Msh3 (MutS Homolog 3), a DNA mismatch repair protein (MMR), was the most highly enriched H2A T101 GlcNAcylated nucleosome interactor along with Msh2 (MutS Homolog 2), another MMR protein, amongst the top seven enriched interacting partners. The Msh2-Msh6 heterodimer has been implicated in nucleosome remodelling that leads to disassembly of the nucleosome^28^. Together these proteomic results provide evidence of increased accessibility for proteins from a relevant cellular sample to corresponding regions of DNA (for example, USF1 or USF2 to E-Box) facilitated by GlcNAcylation, consistent with the open state resulting from nucleosome destabilization suggested by the *in vitro* biophysical and DNAse sensitivity analyses. They also suggested the putative nucleosome remodellers Msh2 and Msh3 as potential partners that might accelerate nucleosome disassembly upon H2A-T101 *O*-GlcNAcylation.

## Discussion

The destabilization of H2A/B dimers in nucleosomes is proposed as an important mechanism to facilitate transcription^29^,^30^. In one mode, the stability of H2A/B dimers in the nucleosome can be regulated by incorporation of histone H2A variants^31^; for example, labile histone H2A variants (H2A.Z.2.2 and H2A.Bbd) have been reported^16^,^32^,^33^ that do not form stable octamers without DNA, but do form nucleosome-like species in the presence of DNA, albeit with lower stability. Reduced stability for H2A.Bbd was attributed^33^ to changes in the dimer–tetramer docking domain, an interface that notably includes the H2A-T101 site that is the focus of this study. We propose here that GlcNAcylation may regulate nucleosome stability in a post-translational enzyme-mediated manner complementary to co-transcriptional histone variant incorporation, where, in essence, GlcNAcylation generates a labile variant *in situ*. Intriguingly, a third complementary PTM-mediated regulation of the tetramer-dimer interface, which employs a different tetramer-alteration mechanism, has been proposed for H4-K91 acetylation and H4-K91 ubiquitinylation^34^,^35^. In this third ‘H4-alteration model' the same or a similar interface may be modulated but with a vital difference (and hence different mechanistic manifold for exploitation in regulation): in this case the core tetramer would be altered to encourage dissociation of the H2A/H2B dimer (the H4-alteration model), whereas our results suggest a H2A-alteration model that would instead ‘trap' an unbound H2A/H2B dimer state. Notably consistent with this, upon *in vitro* OGT treatment, Thr101 modification is observed in isolated H2A, but not when H2A is present in octamers or nucleosomes^5^, indicating that the enzymatic *O*-GlcNAcylation occurs selectively on histone H2A in a ‘non-bound' state.

Based on these combined observations, we propose the following model connecting OGT recruitment to transcriptional regulation (Fig. 3b). H2A/B dimers are in exchange with a free H2A/B pool, especially in transcriptionally active regions where nucleosomes have to be disassembled every time the RNA polymerase passes through^36^. In the presence of OGT, free H2A/B dimers could be O-GlcNAcylated. Speculatively, this may be enabled or enhanced by selective OGT recruitment (Supplementary Fig. 15). This would result in decreased nucleosome stability at these sites upon incorporation of O-GlcNAcylated H2A, thus providing an open chromatin state and which could increase binding of additional chromatin modifying complexes and transcription factors (such as those identified in our quantitative proteomic analyses, vide supra) or by reinforcing transcription by lowering the nucleosome barrier for RNA polymerase passage. These proposals are supported by the observation that OGT recruitment to TSS overlaps very well with DNAse I sensitivity at these promoters and by the direct identification of potential partners (for example, USF1 and USF2) from cellular samples. In addition, reduced nucleosome stability might liberate binding sites for other DNA-binding proteins, such as the MMR proteins also identified from cellular samples. It is, of course, important to note that any modification that destabilizes the nucleosome core would be expected to enrich DNA-binding proteins in this way. We also suggest that this mechanism complements both co-transcriptional H2A-variation (H2A.Z.2.2 and H2A.Bbd) and post-translational H4-alteration (H4-K91 acetylation/ubiquitinylation) to provide a third post-translational H2A-alteration/trapping mechanism. In this way, *O*-GlcNAcylation by OGT presents a potential catalytic mechanism for the amplification of transcriptional activation. In this speculative model, DNA-bound tetramer could remain fixed to a chromatin region thereby allowing such a low abundance modification to play a significant mechanistic role. Additionally, GlcNAcylation could either occur on a transiently ‘open' nucleosome (likely under a Curtin–Hammett regime, still DNA-bound), or act on sufficient of the local free dimer pool to maintain the destabilizing effect within a defined local region. The feasibility of such a mechanism (which is speculative) is clearly subject to caveats of suitable kinetics/lifespan of GlcNAcylation and mobility of any putative free dimer pool. Although the results suggest that OGT may be directly involved in transcriptional regulation, other processes, for example, in histone processing, recycling and localization, could be modulated by *O*-GlcNAcylation. Indeed, a recent report has revealed that *O*-GlcNAcylation can occur co-translationally in some proteins^37^, although it is currently unknown if this also happens for T101 *O*-GlcNAcylation. The exact role of OGT in all of these processes necessitates additional studies addressing the *in vivo* function of this and other reported *O*-GlcNAcylation sites.

## Methods

### Histone and DNA preparation

Histone plasmids (pET3) encoding for canonical *Xenopus laevis* histones were a kind gift from Dr Rob Klose, Department of Biochemistry, Oxford. The H2A T101C mutation was introduced using the Quik-Change mutagenesis kit (Agilent; #200518) and was verified by sequencing. H2A_T101C_F: ctgctcggaagagtctgtatcgctcagggcgg; H2A_T101C_R: ccgccctgagcgatacagactcttccgagcag.

Histone proteins were produced in *E. coli* BL21 (DE3) pLysS as inclusion bodies and purified by size-exclusion (Superdex S200) and ion-exchange (Hitrap SP HP) chromatography^10^,^12^. BL21(DE3) pLysS cells transformed with the appropriate plasmid were grown at 37 °C in 2xTY medium containing ampicillin and chloramphenicol at 37 °C until OD600=0.4–0.6. Expression was induced by addition of IPTG (0.5 mM) and continued at 37 °C for 2 h (3 h for Histone H4). Cells were harvested by centrifugation and the pellet was re-suspended in lysis buffer (50 mM TRIS pH 7.5, 100 mM NaCl, 1 mM EDTA and 5 mM BME) with protease inhibitor (one tablet per 50 ml, Roche, 11697498001, 40 ml total) and frozen and stored at −80 °C. Frozen cells were thawed in a water bath, lysed by sonication and inclusion bodies were collected by centrifugation. The supernatant was discarded and the pellet was re-suspended in 40 ml TW buffer (lysis buffer+1% Triton X) and inclusion bodies were again collected by centrifugation. The pellet was washed two more time with TW buffer and once with wash buffer. 7–15 ml (for 1–2 l culture) unfolding buffer (7 M guanidinum hydrochloride, 10 mM TRIS pH 7.5, 1 mM EDTA, 10 mM DTT, 1 mM benzamidine) was then added, and shaken for 1 h at room temperature (rt), insoluble materials were removed by centrifugation. The solubilized histones were collected and loaded onto an S200 size-exclusion column (equilibrated with SAU-1000 buffer; 7 M Urea, 20 mM NaOAc pH 5.2, 1 M NaCl, 1 mM EDTA and 5 mM BME). The histones were eluted with SAU-1000 buffer, and analysed by SDS–PAGE. Fractions containing histones were pooled dialysed against water containing 2 mM BME, and lyophilized. Histones were further purified by IEX using a HiTrap SP column. The lyophilized histones were dissolved in SAU-200 buffer (like SAU-1000 but with 200 mM NaCl) for 15 min at 4 °C and then loaded onto a HiTrap SP HP (5 ml, GE Healthcare) column equilibrated in SAU-200 buffer. Histones were eluted with a gradient from 0–100% SAU-600 buffer (like SAU-1000 but with 600 mM NaCl) in 20 CV. The fractions containing pure histones were pooled dialysed against water with 2 mM BME, and lyophilized. Lyophilized histones were stored at −80 °C.

The plasmid encoding for the 145 bp 601 DNA construct^15^ was a kind gift from Jinrong Min (SGC Toronto). Large-scale DNA preparation was performed essentially as described by Dyer *et al.*^12^ The 601 DNA plasmid (pUC 57) was transformed into HB101 cells, which were plated on ampicillin (100 μg ml^−1^) containing LB-Agar plates and grown overnight. The next morning, two 15 ml pre-cultures (2xTY containing ampicillin at the same concentration) were inoculated with one colony each and grown at 37 °C until slightly turbid (4–5 h). These where then used to inoculate four flasks (700 ml TB with ampicillin at the same concentration). These were shaken at 37 °C for 22 h. The cells were then harvested (7,000 r.p.m.; 7 min; F10BA-6x500y rotor) and resuspended in a total of 120 ml alkaline lysis buffer 1 (50 mM glucose, 25 mM Tris pH 8.0 and 10 mM EDTA) and split into two 500 ml centrifugation tubes (60 ml per tube). To each tube 120 ml alkaline lysis buffer 2 (0.2 M NaOH, 1% (w/v) SDS) was added and the mixture was shaken and incubated on ice for 10–20 min. Then 210 ml of cold alkaline lysis buffer 3 (4 M sodium acetate, 2 N acetic acid) was added and the mixture was shaken to mix and subsequently incubated on ice for 20 min. The mixture is then centrifuged (1,000*g*, 20 min, 4 °C) and filtered through tissue paper. Then 0.52 v/v isopropanol is added and the mixture is incubated at rt for 15 min and then centrifuged (1000*g*, 30 min, 20 °C; in polypropylene tubes; Beckman Coulter 41121703). The supernatant was then decanted and the pellet transferred to a 50 ml Falcon tube. Then 20 ml TE 10/50 and 21 μl RNase A (100 mg ml^−1^; Qiagen; 19101) was added and the pellet was dissolved by shaking at 37 °C overnight. Then the mixture was centrifuged (JA25.50; 2000, r.p.m.; 15 min; 4 °C) to remove particulates. To the supernatant one-fifth of 4 M NaCl and two-fifth 40% PEG 6000 was added to precipitate the DNA. The mixture was shaken at 37 °C for 5 min, incubated on ice for 30 min and centrifuged at 3000*g* for 20 min. The supernatant was decanted and the pellet was dissolved in 15 ml TE 10/0.1 and the pellet was dissolved by shaking at 37 °C (2–3 h). The DNA concentration was then determined and 30 units of EcoRV (NEB; R0195) per nmol EcoRV site were added. The reaction was incubated at 37 °C for 16 h and completion was checked by agarose gel. If the reaction is complete, the plasmid is precipitated by addition of 0.18 vol 4 M NaCl and 0.31 vol 40% PEG 6000. The mixture is incubated on ice for 1 h and the centrifuged (JA25.50, 27,000*g*, 20 min, 4 °C). The supernatant is decanted and the insert is recovered by addition of 3 vol EtOH, incubation on ice for 1 h and centrifugation (JA25.50, 27000*g*, 20 min, 4 °C). The insert is dissolved in 3–4 ml TE 10/0.1 and purified by phenol extraction and ethanol precipitation.

### Denaturing ESI-MS

The chemical protein modification was monitored using LC-MS. LC-MS was performed using a Micromass LCT (ESI-TOF-MS) and a Shimadzu Prominence HPLC equipped with a Dionex Proswift RP-4H column. The column was run at 0.4 ml min^−1^. Solvents used were water with 0.1% formic acid and 95:5 Acetonitrile: water with 0.1% formic acid. The proteins were separated using a gradient from 5% B to 95% B in 4 min. After this the column was washed for 2 min with 95% B and then equilibrated with 5% B for 2.5 min. The electrospray source was set to 3200 V capillary voltage and 25 V cone voltage.

Data were analysed using Masslynx and calibrated using myoglobin. Deconvolution was performed using the maximum entropy algorithm.

### H2A T101Dha synthesis

The reaction was performed in denaturing reaction buffer (RB; 5 M Gd.HCl, 50 mM TRIS pH 8.0). An amount of H2A T101C (10 mg) was weighed into an Eppendorf tube and dissolved in 500 μl RB. Then 15 mg DTT was added and the protein was shaken at rt for 30 min. Excess DTT was removed by using a PD minitrap column (in RB) giving a final concentration of 10 mg ml^−1^ H2A T101C (Supplementary Fig. 1).

To 900 μl of reduced H2A T101C (10 mg ml^−1^; 0.74 mM) was added 100 μl of a DBHDA stock solution (0.5 M in DMF; 50 mM final; 90 eq). The reaction was shaken at rt for 30 min and then at 37 °C for 4 h. The reaction was monitored by LC-MS and after completion excess reagent was removed using a PD minitrap column (in RB, loaded with 500 μl). After 4 h at 37 °C full conversion of the cysteine containing starting material to Dha was observed (Supplementary Fig. 2).

### H2A T101 GlcNAc synthesis

To 2 ml H2A T101Dha (4 mg ml^−1^, 270 μM) was added GlcNAc-SH (120 mg, 505 μmol, 250 mM final, 925 eq) and the reaction mixture was shaken at 37 °C for 3.5 h after which time full conversion to H2A T101GlcNAc was observed by LC-MS. Excess GlcNAc-SH was removed by using a PD10 desalting column (in RB) and the resultant product was stored at −20 °C for dimer reconstitution (Supplementary Fig. 3).

### Complex reconstitution

Histone complex reconstitution was performed as described^12^,^38^,^39^. For the reconstitution, histones (powder after freeze-drying; 3–4 mg of each histone) were dissolved in 2 ml unfolding buffer (7 M Gd.HCl, 10 mM TRIS pH 7.5, 1 mM EDTA, 1 mM DTT) by rotating at 4 °C for 30 min. Chemically GlcNAcylated H2A was used after the modification reaction in RB. The protein concentration was then measured using a Nanodrop-1000. Wt octamers were reconstituted by combining a 1/1/1.1/1.1 ratio of H3/H4/H2A/H2B. For reconstitution of GlcNAcylated or wt H2A/B dimers equal amounts of each protein were combined. Wt H3/H4 tetramers were refolded by mixing equal amount of each protein. The solution was adjusted to a final concentration of 1–1.5 mg ml^−1^ and dialysed into 3 charges of 2 l refolding buffer (2 M NaCl, 10 mM TRIS, pH 7.5, 1 mM EDTA, 5 mM BME) for at least 4 h each. Precipitated protein was removed by centrifugation and the refolded histones were concentrated to 1–1.5 ml and purified by gel filtration (Superdex S200, 16/60, in refolding buffer). For octamer reconstitution from dimers and tetramers, the indicated dimer and tetramer ratio was mixed in refolding buffer, incubated on ice for 1 h and subsequently purified by gel filtration as above. For the analysis of octamer reconstitutions using different tetramer/dimer ratios (Supplementary Fig. 5) the size-exclusion analysis was performed using a Superdex S200 10/300 GL in refolding buffer.

### Nucleosome reconstitution by salt gradient dialysis

Wt nucleosomes were reconstituted by combining wt octamer and DNA in a 1: 0.9 molar ratio. GlcNAcylated nucleosomes were reconstituted by combining dimer: tetramer: DNA in a 2: 1: 0.9 ratio. For dimer titrations the amount of dimer was varied, while the tetramer and DNA amount was kept constant. The mixtures were then transferred into a 6–8 kDa MWCO dialysis tube. The solution in dialysis tubing was dialysed against either a continuous gradient from high-salt buffer (2 M KCl, 10 mM Tris pH 7.5, 1 mM EDTA, 1 mM DTT) to low-salt buffer (250 mM KCl, 10 mM Tris pH 7.5, 1 mM EDTA, 1 mM DTT over 36 h) for large-scale reconstitution or against a series of step gradients for micro-scale reconstitution as described^12^. After completion of the gradient the refolded nucleosomes were dialysed two times against no-salt buffer (10 mM TRIS pH 7.5, 1 mM EDTA, 1 mM DTT)

### CD analysis of the H2A/B dimers

CD spectra were recorded using a Chirascan CD-spectrophotometer (Applied Photophysics). H2A/B dimers were buffer exchanged into 20 mM NaHPO_4_, pH 6.7, 1 mM EDTA, 150 mM NaCl by viva-spin. A final concentration of 10 μM dimer in 500 μl in a micro cuvette (1 mm path length) was used for each measurement. CD spectra were recorded from 200–300 nm with a step size of 1 nm and 0.5 s per point. The initial spectra were recorded in triplicates. The three traces were averaged and smoothed using a moving average (window size=4) and the background was subtracted. For melting curves the temperature was increased stepwise from 25–90 °C in 1 °C steps with a hold time of 60 s. The temperature of the sample as measured by a sample probe was recorded for each step. The raw data were exported from the Pro-data as CSV and further processed using OriginPro. The melting temperature was extracted by plotting the CD at 220 nm against temperature and fitting using a Boltzmann sigmoidal function. Reported are the fitted parameters including the standard error.

### Non-denaturing PAGE analysis

‘Native' PAGE analysis was performed using Novex TBE Gels (6%, 10-well, Invitrogen, EC6265BOX). The gel was prerun in 0.5 × TBE at 150 V for 90 min at 4 °C. Then 1–4 nmol of nucleosome in 5 μl, supplemented with 1 μl 30% sucrose as loading buffer, was added per well. The gel was run at 4 °C at 150 V for 90 min and subsequently stained with SYBR Gold (Invitrogen, S-11494) and visualized using a Bio-Rad Gel Doc XR+ machine.

### DSF melting temperature analysis

DSF analysis was performed on a Bio-Rad MJ Mini real-time PCR machine using the Opticon Monitor 3 software. For the analysis 23 μl of 2.5 μM nucleosome in 10 mM NaHPO_4_, pH 7.5, 1 mM EDTA, 150 mM NaCl and 2 μl of 25 × SYPRO Orange (diluted from 5,000 × Concentrate in DMSO; Life technologies S-6650) were added into a real-time PCR plate and sealed with transparent film. Then the sample was first kept for 1 min at 15 °C and then heated from 15–95 °C in 1 °C steps with one minute at each temperature. Fluorescence was read out for each temperature. The data was analysed using the excel sheets described by Niesen *et al.*^18^ Melting temperatures were extracted by fitting using a Boltzmann sigmoidal function in OriginPro. Reported are the fitted parameter including the s.e.m.

### CD analysis of the nucleosomes

Nucleosomes (0.62 μM) were buffer exchanged into 10 mM NaHPO_4_, pH 7.5, 1 mM EDTA, 150 mM NaCl. For the measurement 180 μl of nucleosome solution in a micro cuvette (1 mm path length) was used. CD spectra were recorded from 200–300 nm with a step size of 0.5 nm and 1 s measurement per point. Temperature was ramped stepwise from 25–90 °C in 1 °C steps with 120 s at each temperature. Data was processed as described above for the dimer.

### Non-denaturing ‘Native' MS

Nucleosomes were buffer exchanged using Micro Bio-Spin chromatography columns (Bio-Rad) equilibrated with 50 mM ammonium acetate pH 7.5. Spectra were acquired on a QToF II MS (Waters) modified for high mass analysis and preservation of non-covalent interactions^40^. Aliquots (3 μl) diluted to 1–2.5 μM were introduced into the mass spectrometer from gold-plated capillary needles (Harvard Apparatus). Capillary voltages were 1.4–1.6 kV, cone voltages were 80–100 V and collision voltage was 10 eV. ToF pressure 2.05 × 10^−6^ bar, analyser pressure 1.54 × 10^−4^ bar and backing pressure 4.26 × 10^−3^ bar. The recorded mass spectra were calibrated externally using caesium iodide in water. Data were acquired and processed with Mass Lynx V4.1 software (Waters) with minimal smoothing and no background subtraction.

### ChIP-Seq and DNase I sensitive site analysis

OGT ChIP data published by Vella *et al.*^21^ was used for the analysis. DNase I sensitivity data were obtained from data deposited by the ENCODE project (Digital DNaseI from ENCODE/ University of Washington; ES-E14; 129/Ola; E0; hotspot-broadPeak: wgEncodeUwDnaseEse14129olaME0HotspotsRep1)^22^,^41^. Previously called peaks reported in Vella *et al.*^21^ and the DNaseI hotspot-broadPeak were used to analyse overlap between OGT and DNaseI sensitive sites. Data was analysed using Galaxy^42^,^43^,^44^ and visualized using the UCSC genome browser^45^.

Overlapping regions were found using the ‘Operate on Genomic Intervals/intersect' tool in Galaxy. For 11552 OGT peaks 11233 overlap with DNase-sensitive sites (97%). For DNase-sensitive sites 12257 of 270487 peaks overlap with OGT peaks (4.5%). Further statistical analysis was performed using The Genomic HyperBrowser (https://hyperbrowser.uio.no/)^46^. Using the Statistical analysis tool, we tested for significance of the overlap using following settings: Alternative hypothesis: more, Null model: Preserve segments (T2) and segment length (T1); Randomize Positions (T1) (MC), MCFDR sampling depth: Moderate resolution of global and local *P*-values, Random seed: Random. With these setting we obtained a *P*-value of 0.000999 for the overlap of OGT (T1) and DNase (T2) sensitive sites.

To analyse peaks that are close at TSS, we included all OGT (4,000) and DNase (12,677) peaks that are within +− 500 bp of an annotated TSS. Of these 3,894 OGT peaks (97%) overlap with DNase-sensitive sites and 3,983 DNase-sensitive regions overlap with OGT peaks (31%). Using the same parameters as described above a *P*-value of 0.000999 was obtained for the overlap of OGT (T1) and DNase (T2) sensitive sites.

### Quantitative MS with H2A T101 GlcNAc-modified nucleosome

The HeLa nuclear extract was prepared using an adapted protocol: http://www.cyclex.co.jp/resource/protocol/classical_biochemical/preparation_of_nuclear_extracts.pdf] Briefly, HeLa cells were grown to near-confluency in a HYPERFlask cell culture vessel (Corning Life Sciences) containing 500 ml DMEM medium supplemented with 10% FBS and 1% Pen-Strep. Cells were trypsinized and were washed with 10 ml of ice-cold PBS buffer by centrifugation at 300*g* for 5 min at 4 °C. Cells were re-suspended in 10 ml of lysis buffer (10 mM Tris pH 7.5, 10 mM NaCl, 15 mM MgCl_2_, 250 mM Sucrose, 0.1 mM EGTA, 0.5% NP-40 with complete protease and phosphatase inhibitors) and vortexed for 10 s. Cells were incubated on ice for 15 min followed by being spun through 40 ml of sucrose cushion (30% Sucrose, 10 mM Tris pH 7.5, 10 mM NaCl, 3 mM MgCl_2_ with complete protease and phosphatase inhibitors) at 1300, g for 10 min at 4 °C. The supernatant was discarded. The isolated nuclei were washed with 10 ml of ice-cold wash buffer (10 mM Tris pH 7.5, 10 mM NaCl with complete protease and phosphatase inhibitors). Nuclei were re-suspended in 1 ml of extraction buffer (50 mM HEPES pH 7.9, 420 mM NaCl, 0.5 mM EDTA, 0.1 mM EGTA, 10% Glycerol, 0.1% NP-40 with complete protease and phosphatase inhibitors) and sonicated for 30 s. The ruptured nuclei were incubated on ice for 30 min followed by centrifugation at 13000, g for 10 min at 4 °C. The supernatant, nuclear extract, was collected. The concentration of nuclear extract was determined by BCA assay. The nuclear extract was diluted using a buffer such that final concentration was 0.5 mg ml^−1^ and buffer conditions were: 20 mM HEPES pH 7.9; 150 mM NaCl; 0.2 mM EDTA; 20% glycerol; and 0.1% DTT with complete protease and phosphatase inhibitors (Binding Buffer). The nuclear extract aliquots were flash-frozen in liquid nitrogen and stored at −80 °C.

The plasmid encoding 186 bp ‘601' DNA was a kind gift from Dr Till Bartke, Imperial College, London. The nucleosomal DNA was excised and biotinylated as described^12^,^24^ and further used for reconstitution of wt and modified nucleosome as described earlier. Reconstituted nucleosomes were analysed using native PAGE.

Interaction studies between the nucleosome and the nuclear extract were performed as described above. Briefly, 20 μg of each nucleosome were immobilized separately on 100 μl Dynabeads Streptavidin MyOne T1 (Life Technologies) in the reconstitution buffer (10 mM Tris pH 7.5, 250 mM KCl, 1 mM EDTA, 1 mM DTT with 0.1% NP40). The immobilization efficiency was monitored by measuring the absorption value at 260 nm of the supernatant. Immobilized nucleosome was washed with the binding buffer (20 mM HEPES pH 7.9, 150 mM NaCl, 0.2 mM EDTA, 20% Glycerol, 0.1% NP40, 1 mM DTT with complete protease and phosphatase inhibitors) and then incubated with 1 ml of nuclear extract (0.5 mg ml^−1^ in binding buffer) for 4 h at 4 °C. After stringent washes with binding buffer, the interacting partners were eluted using 100 μl of 8 M Urea, 2 M NaCl.

Tryptic digestion of the interacting partners from each nucleosome sample were performed using the filter-aided sample preparation protocol^47^. Digestion was quenched by addition of 1% formic acid solution. Resulting tryptic peptides were separated using an EASY-nLC 1000 UHPLC system (Proxeon) coupled to a Q Exactive mass spectrometer (Thermo Fischer Scientific). Peptides were trapped on an in-house packed trap column (75 μm i.d. × 20 mm, reprosil C18, 3 μm, 120 Å) and separated on an EASY-spray Acclaim PepMap analytical column (75 μm i.d. × 500 mm, RSLC C18, 2 μm, 100 Å). Samples were loaded at a pressure of 500 bar with 100% solvent A (0.1% formic acid, 5% DMSO in water) and the peptides separated by a linear gradient (length: 120 min, 8–28% solvent B (0.1% formic acid, 5% DMSO in acetonitrile), flow rate: 200 nl min^−1^). The separated peptides were electrosprayed directly into the Q Exactive operated in a data-dependent mode through an EASY-Spray nanoelectrospray ion source. Full-scan MS spectra were acquired in the Orbitrap (scan range 350–1500 *m/z*, resolution 70,000, AGC target 3e6, maximum injection time 100 ms). After the MS scans, the 20 most intense peaks were selected for HCD fragmentation at 30% of normalized collision energy. HCD spectra were also acquired in the Orbitrap (resolution 17,000, AGC target 5e4, maximum injection time 120 ms).

The raw data files generated were processed using MaxQuant software (Version 1.4.3.17), integrated with Andromeda search engine^48^,^49^. For protein groups identification, peak lists were searched against human database (Swiss-Prot, version 04/13) as well as list of common contaminants by Andromeda. Trypsin with a maximum number of missed cleavages of 2 was chosen. Acetylation (protein N-term) and oxidation (methionine) were used as variable modifications, while carbamidomethylation (Cysteine) was set as a fixed modification. Protein and PSM false discovery rate were set at 0.01. Label-free quantification (LFQ) was performed using the in-built algorithm in the MaxQuant^26^. LFQ intensities were uploaded into Perseus software (Version 1.4.2.27) for further analysis. Contaminant, reverse and protein groups only indentified by site were filtered off. Protein groups identified in both bait and control samples and each replicates were used. Their LFQ intensities were logarithmized and mean value was calculated. The difference between the average LFQ intensities of protein groups between bait and control samples alongside total intensity were evaluated by Significance B to generate a *P*-value.

### Salt-based disassembly of the nucleosome

For salt-based disassembly^16^,^50^, to 5 μl of each nucleosome (4 μM in 10 mM Tris pH7.5, 1 mM EDTA) was added 1 μl of a KCl stock solution to give the final salt concentration as indicated. The samples were then incubated at 37 °C for 60 min and analysed by non-denaturing (‘native') PAGE.

### Thermal disassembly of the nucleosome

10 μl of each nucleosome (4 μM in 10 mM Tris pH7.5, 20 mM KCl, 1 mM EDTA) were incubated at 63, 66, 69, 72 or 75 °C in a PCR machine for 30 min. The samples were centrifuged and the supernatant was analysed using Native PAGE and SDS–PAGE. SDS–PAGE analysis was performed using 12% Bis-Tris gel (Novex). The gel was run in 1 × MOPS buffer at 200 V for 50 min. The gel was stained with InstantBlue (Expedeon).

## Additional information

**How to cite this article:** Lercher, L. *et al.* Generation of a synthetic GlcNAcylated nucleosome reveals regulation of stability by H2A-Thr101 GlcNAcylation. *Nat. Commun.* 6:7978 doi: 10.1038/ncomms8978 (2015).

## Supplementary Material

Supplementary Figures Supplementary Figures 1-16

Supplementary Data 1 Identification and quantification of interacting proteins for H2A T101 GlcNAc modified nucleosome versus wild-type nucleosome
