# Supplementary material for: Generation of a synthetic GlcNAcylated nucleosome reveals regulation of stability by H2A-Thr101 GlcNAcylation
Source: Nat Commun. 2015 Aug 25;6:7978. doi: 10.1038/ncomms8978 (PMC4560749; doi:10.1038/ncomms8978)
Supplement: Supplementary Figures — 1-16 [file ncomms8978-s1.pdf]

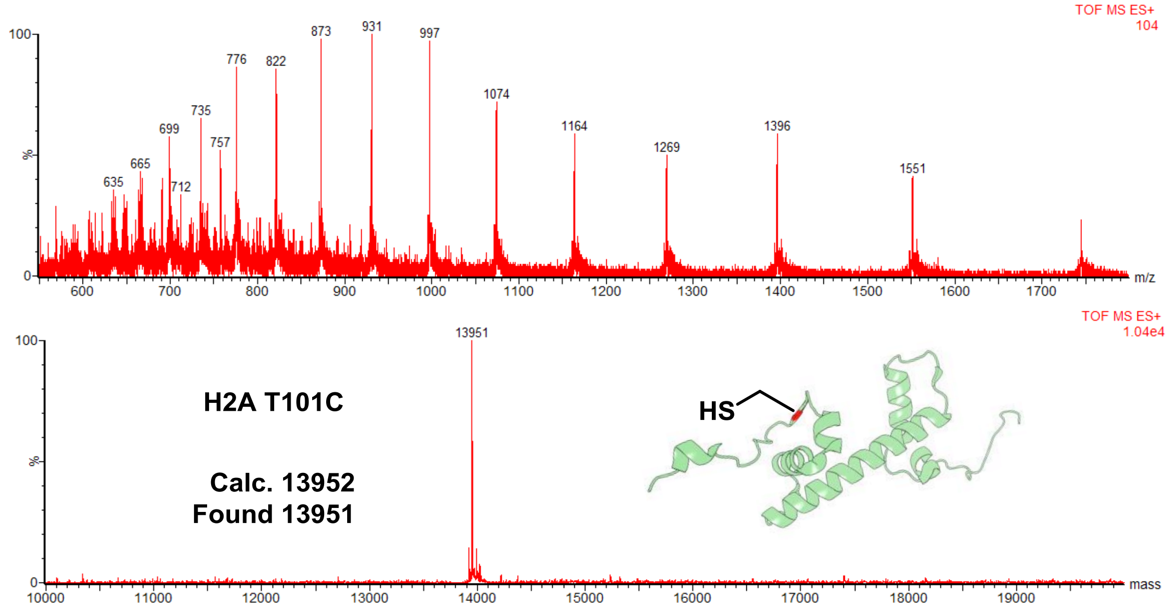

Supplementary Figure 1. LC-MS analysis of the reduced H2A T101C.

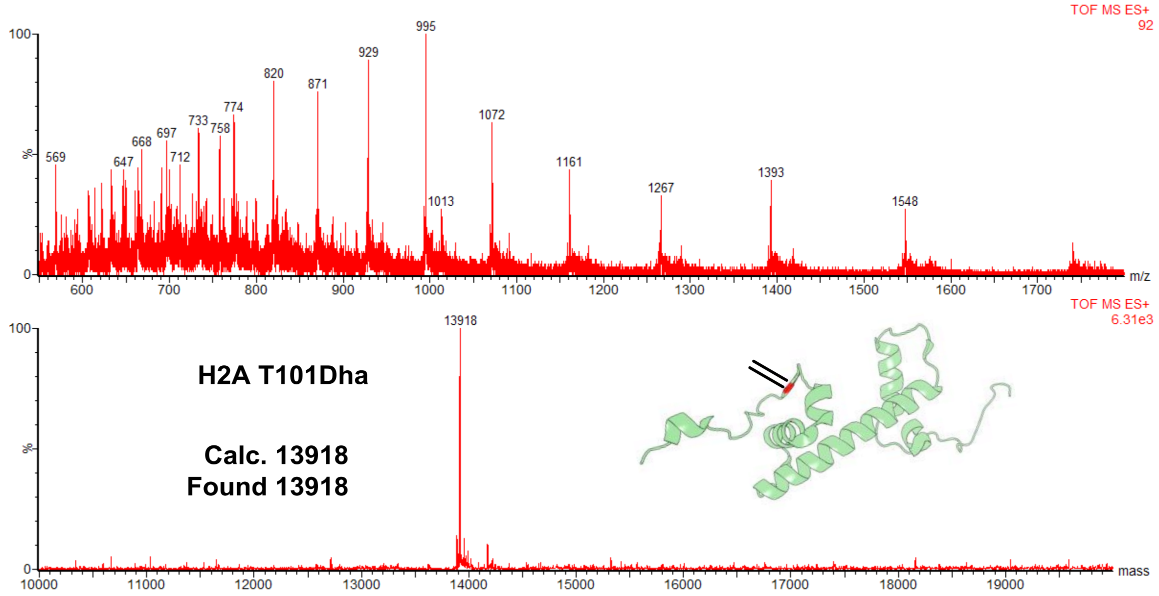

Supplementary Figure 2. LC-MS analysis of H2A T101Dha.

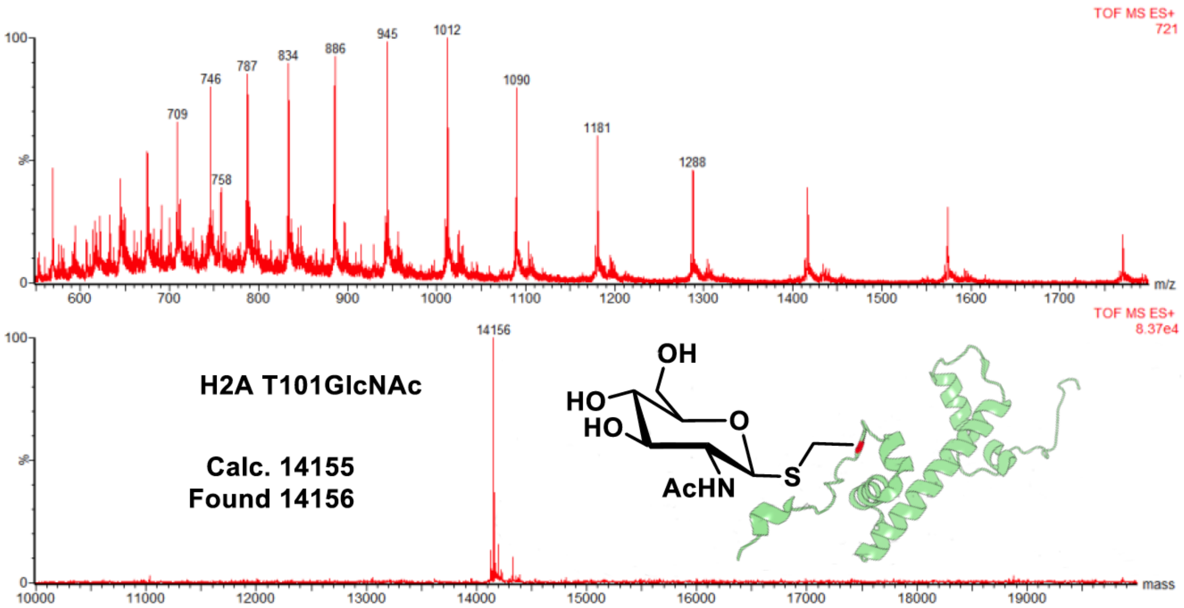

Supplementary Figure 3. LC-MS analysis of H2A T101GlcNAc.

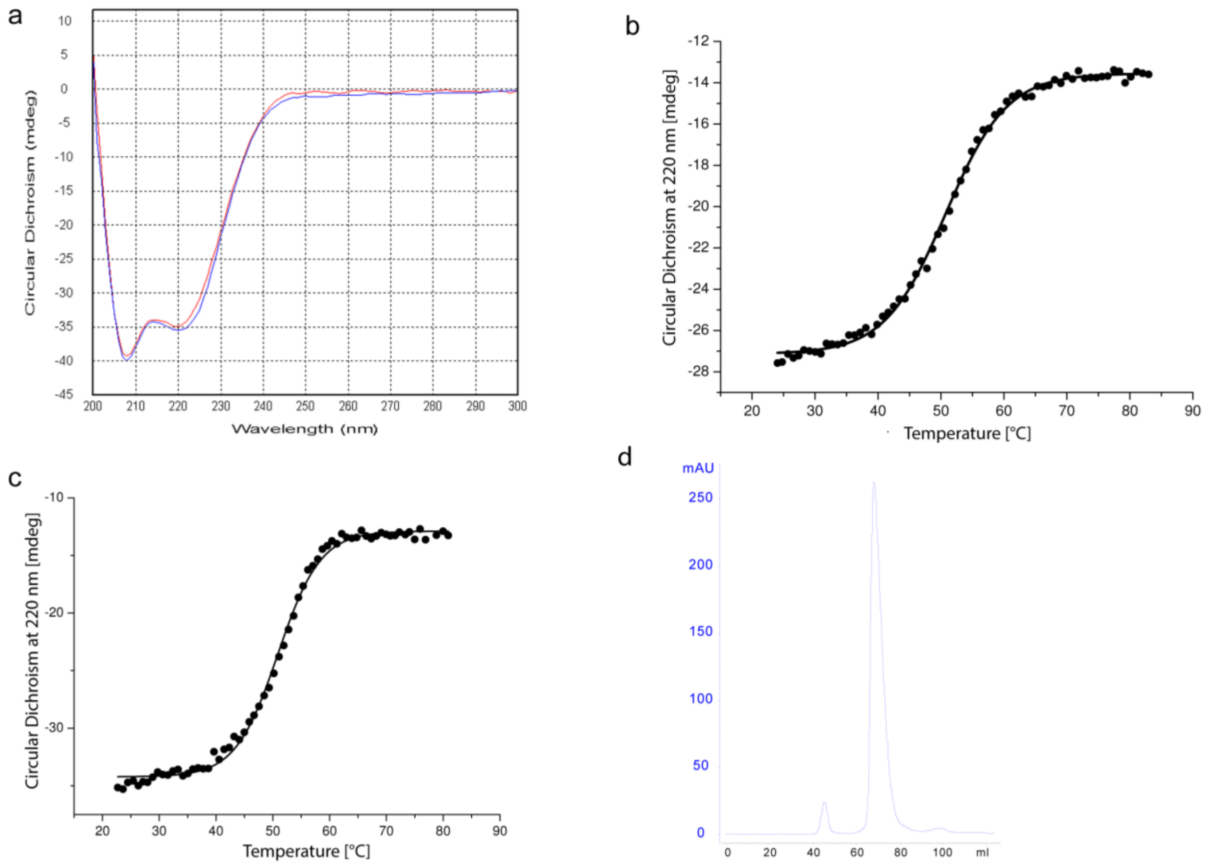

Supplementary Figure 4 CD analysis of wildtype and GlcNAcylated H2A/H2B dimers. **a** Overlay of the CD spectra for wildtype (red) and GlcNAcylated (blue) H2A / H2B dimers. **b** Melting profile of GlcNAcylated H2A / H2B dimer measured by CD. Experiments were performed in triplicates. **c** Melting profile of wildtype H2A / H2B dimer measured by CD. Experiments were performed in triplicates. **d** UV trace of the size exclusion purification of reconstituted wildtype H3/H4 tetramers.

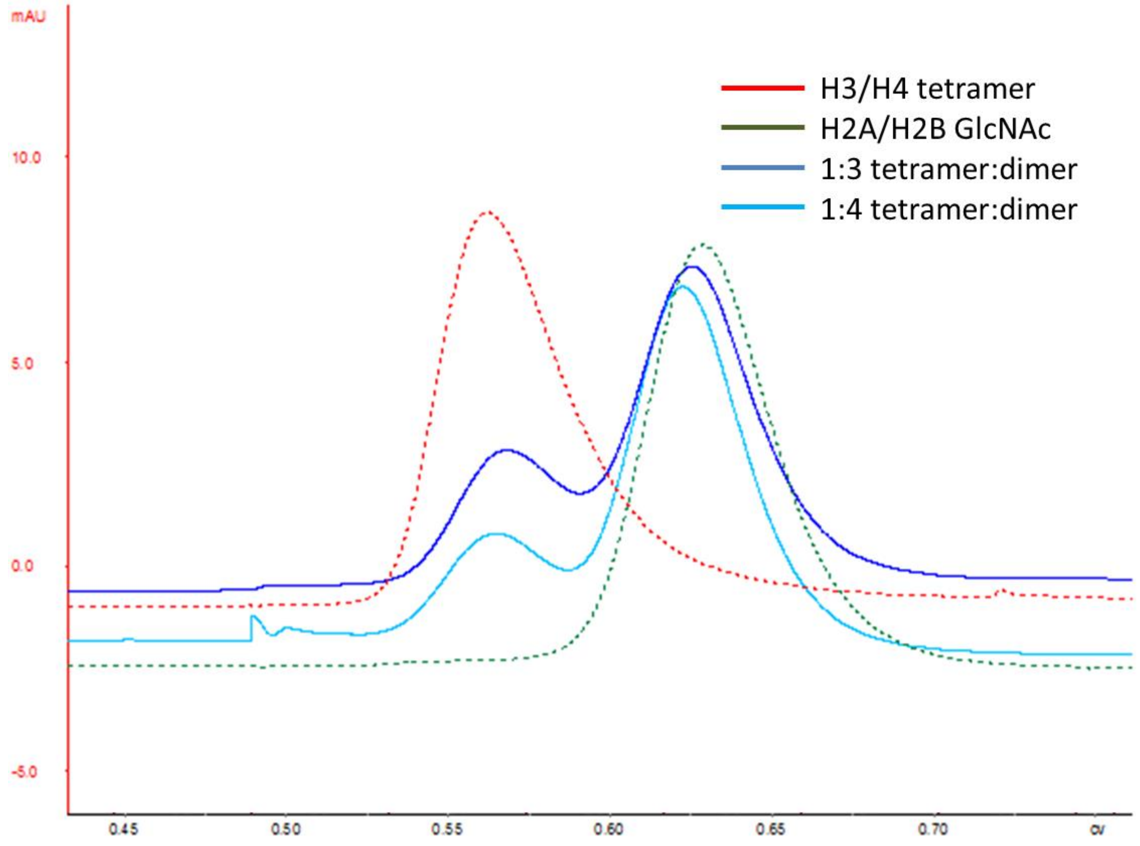

Supplementary Figure 5. Octamer refolding using different H3/H4 tetramer : H2A/H2B-GlcNAc dimer ratios. For reference, SEC traces for the H3/H4 tetramer (red) and the H2A/H2B-GlcNAc dimer (green) are shown.

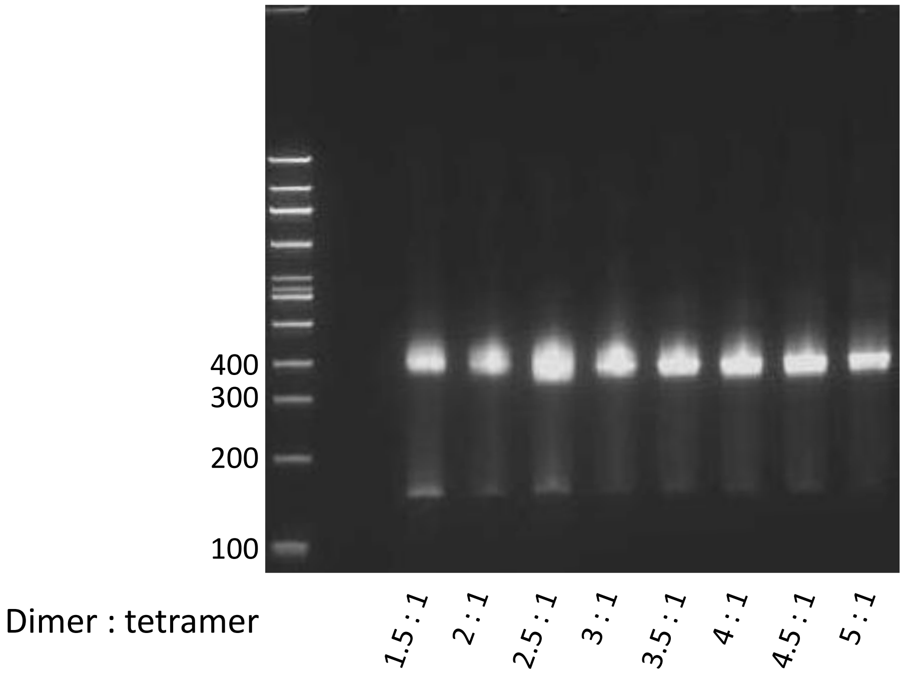

Supplementary Figure 6. Non denaturing PAGE analysis of nucleosome reconstitutions using different H3/H4 tetramer : H2A/H2B-GlcNAc dimer ratios.

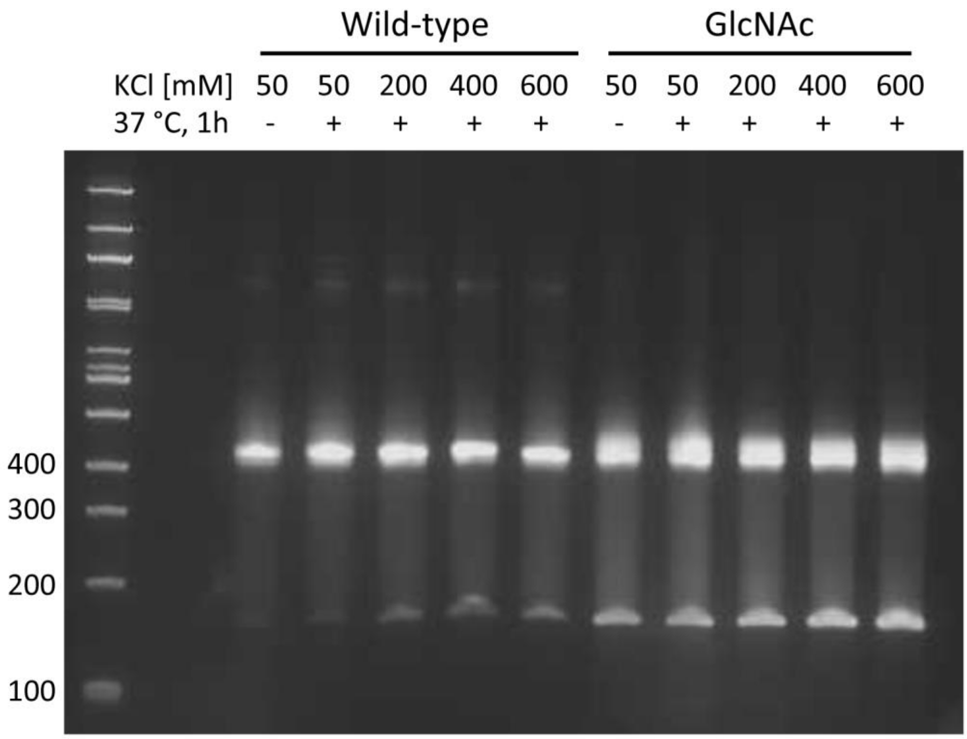

Supplementary Figure 7. Incubation of wt and GlcNAcylated nucleosomes for 1 h at 37°C with different concentrations of KCl.

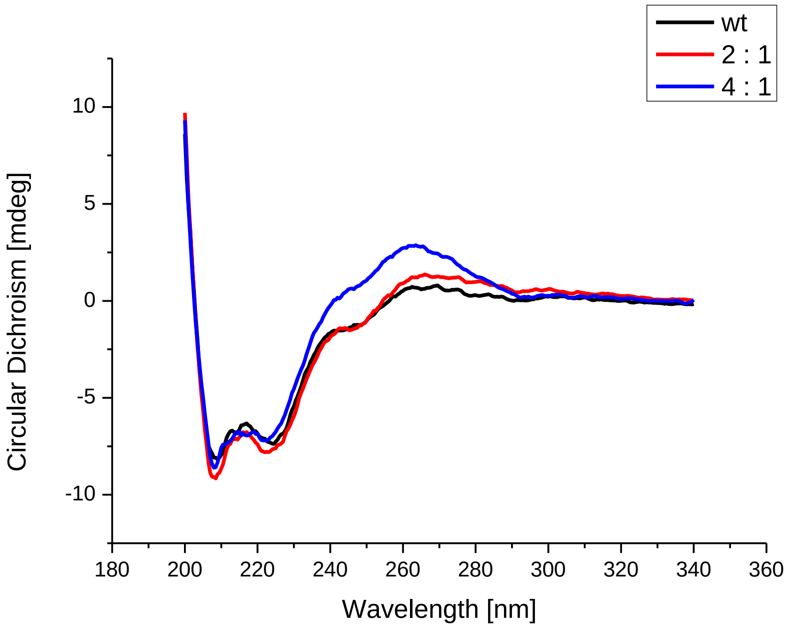

Supplementary Figure 8. CD analyis of wt nucleosomes (black) and nucleosomes reconstituted with different H2A/H2B-GlcNAc dimer : H3/H4 tetramer ratios (2:1, red; 4:1 blue).

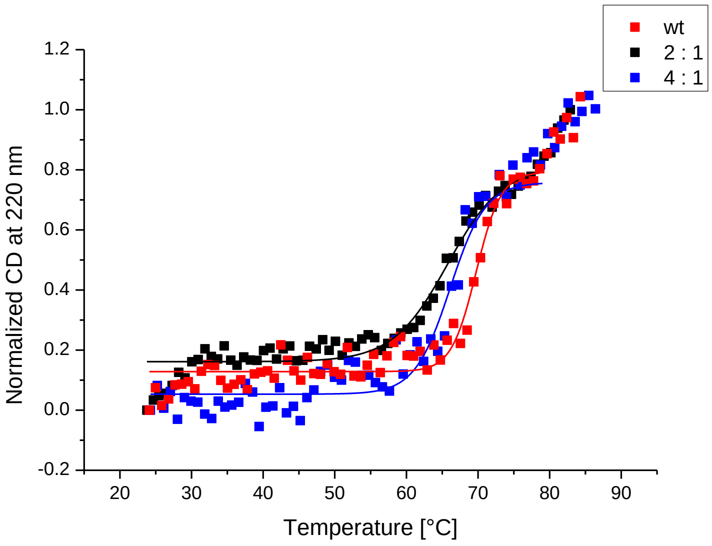

Supplementary Figure 9. Melting profile of wt and GlcNAcylated nucleosomes reconstituted with different dimer : tetramer ratios monitored by CD at 220 nm.

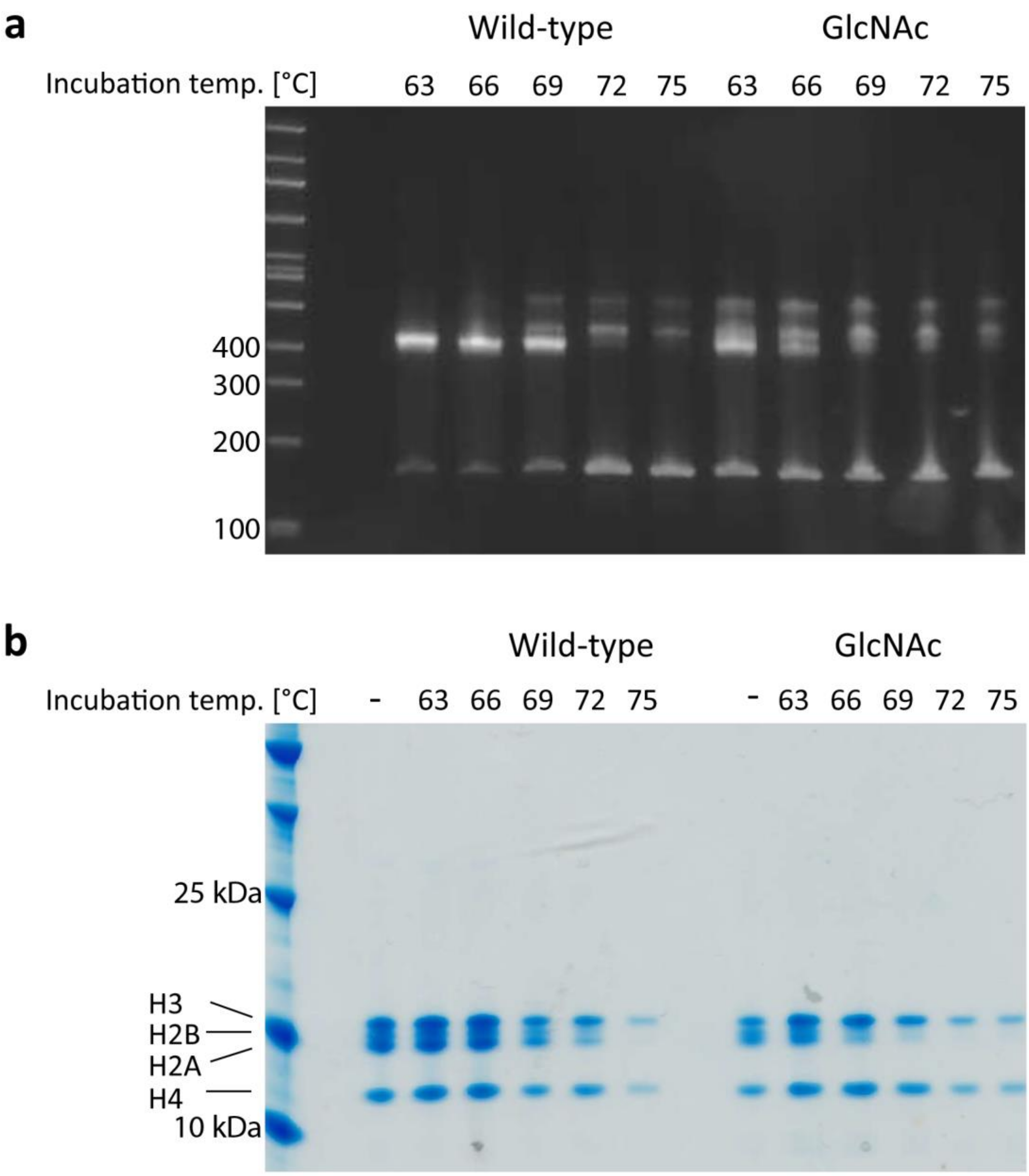

Supplementary Figure 10. Thermal denaturation of wt and GlcNAcylated nucleosomes. **a** Non-denaturing **'**Native' PAGE analysis of nucleosomes incubated at the indicated temperature for 30 min. **b** SDS-PAGE analysis of the supernatant of the thermal disassembly.

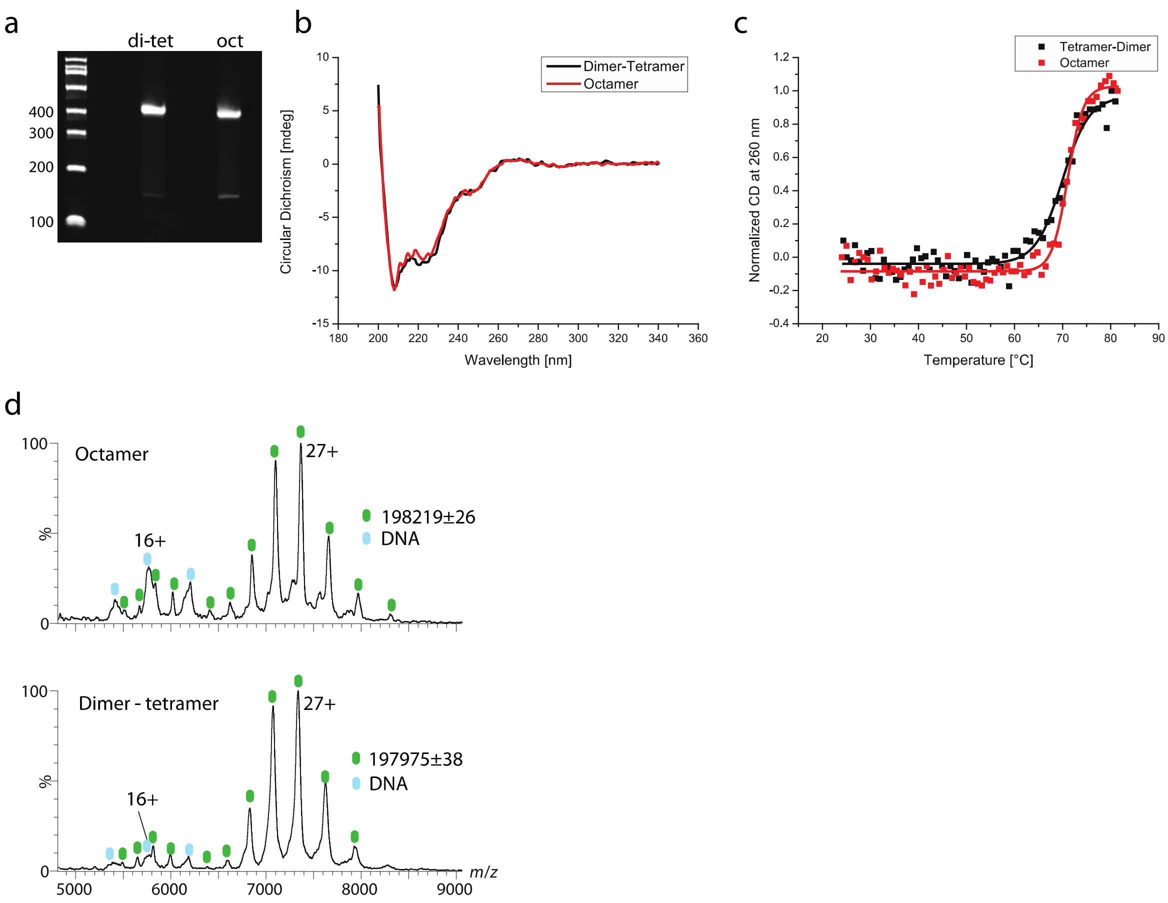

Supplementary Figure 11. Comparison of wt nucleosome reconstituted with wt octamers or wt H2A/B dimers and H3/4 tetramers. **a** PAGE analysis of differently reconstituted nucleosomes **b** Overlay of the CD spectra for nucleosome reconstituted with histone octamers (red) and histone di- and tetramers (black). **c** Melting profiles of nucleosome reconstituted from histone octamers (red) and histone di- and tetramers (black) measured by CD at 260 nm. The melting temperatures of the nucleosomes reconstituted from separate di- and tetramers displayed only a slight reduction in melting temperature (Tm 70.0 ± 0.4 °C; wt: 71.0 ± 0.2 °C). **d** Analysis of differently reconstituted nucleosomes using nESI-MS

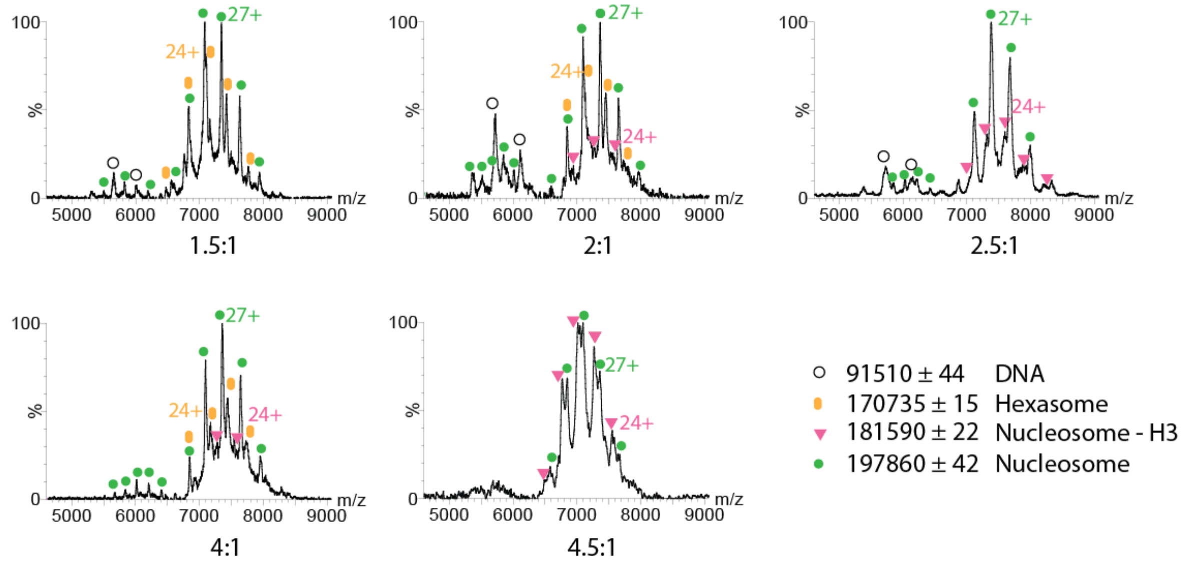

Supplementary Figure 12. Non denaturing MS analysis of nucleosomes reconstituted with different H3/H4 tetramer : H2A/H2B-GlcNAc dimer ratios. The dimer : tetramer ratio is given below the spectrum.

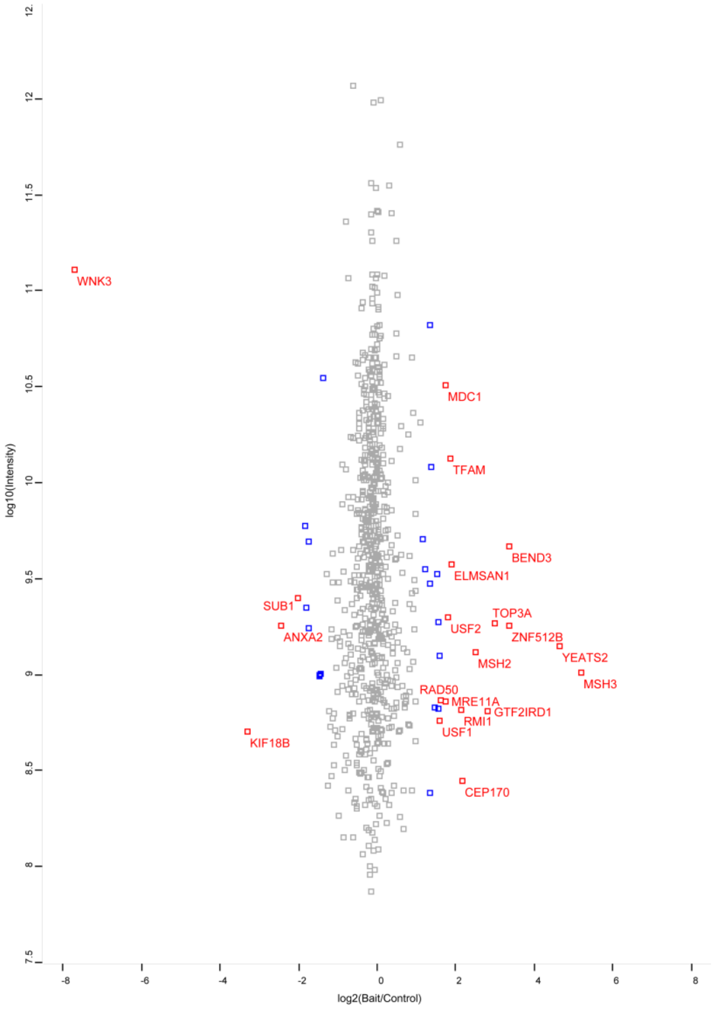

Supplementary Figure 13: Quantitative proteomics identifies preferential interacting partners for H2A T101 GlcNAc nucleosome (bait) over wild-type nucleosome (control) among n=720 identified proteins identified in two independent biological replicates. Significant interactors identified by Label free quantification (LFQ) on Significance B analysis with a Benjamini-Hochberg FDR of 1 % are shown in blue in a scatter plot. Top 20 significant interactors among them are denoted in red with their gene name. Scatter plot: x axis, logarithmized ratio of average LFQ intensities of protein groups in the bait over control pulldown experiment from two independent replicates; y axis, log10-transformed summed up intensity of protein groups.

a

ATCGGATCTTACATGCACAG GATGTATATATCTGA**CACGT**
GCCTGGAGACTAGGGAGTAA TCCCCTTGGCGGTTAAAACG
CGGGGGACAGCGAGTACGTG CGTTTAAGCGGTGCTAGAGC
TGTCTACGACCAATTGAGCG GCCTCGGCACCGGGATTCTC
CAGGGCGGCCGCGTAGTACT GAAUbioUbio

b

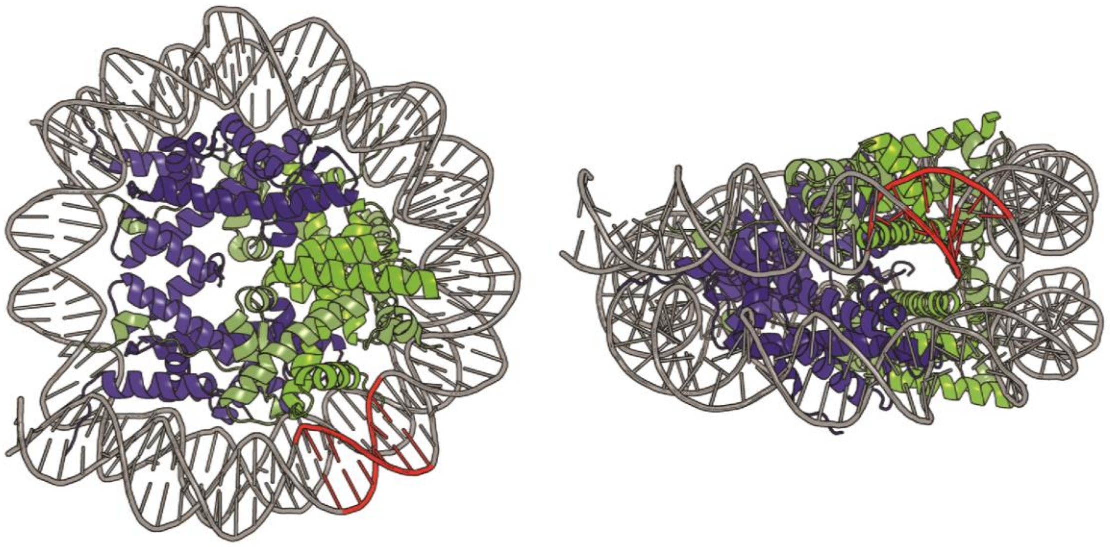

Supplementary Figure 14. Location of the E-Box sequence in the 601 DNA used for nucleosome reconstitution. **a** Sequence of the used 601 DNA with the E-Box sequence marked in red. **b** Location of the E-Box (red) in the 601 DNA in the crystal structure of the nucleosome (pdb: 3LZ0).

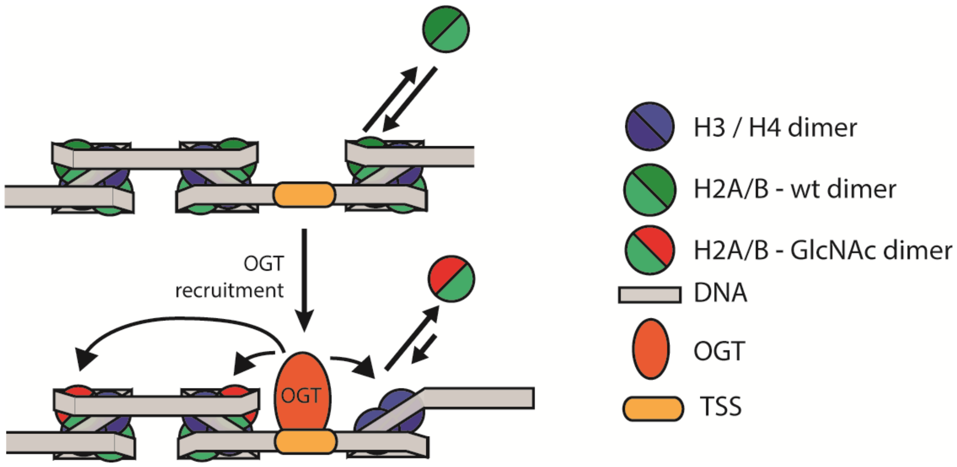

Supplementary Figure 15. A speculative additional role for OGT recruitment in the suggested mechanism of OGT (see also Figure 3 in main text). Localization of OGT is not due to direct DNA binding, but due to association with other chromatin regulating complexes.

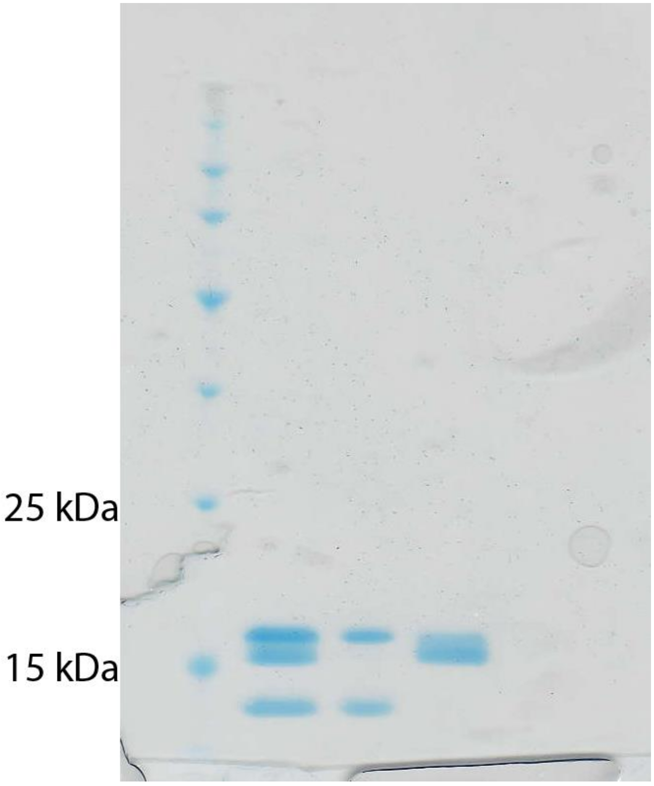

Supplementary Figure 16. Uncropped image used in Fig. 1f
